# A Simple and Rapid Method for Preparing a Cell-Free Bacterial Lysate for Protein Synthesis

**DOI:** 10.1371/journal.pone.0165137

**Published:** 2016-10-21

**Authors:** Nitzan Krinsky, Maya Kaduri, Janna Shainsky-Roitman, Mor Goldfeder, Eran Ivanir, Itai Benhar, Yuval Shoham, Avi Schroeder

**Affiliations:** 1 Laboratory for Targeted Drug Delivery and Personalized Medicine Technologies, Department of Chemical Engineering, Technion – Israel Institute of Technology, Haifa, Israel; 2 The Interdisciplinary Program for Biotechnology, Technion – Israel Institute of Technology, Haifa, Israel; 3 Department of Biotechnology and Food Engineering, Technion – Israel Institute of Technology, Haifa, Israel; 4 Department of Molecular Microbiology and Biotechnology, The Georg S. Wise Faculty of Life Sciences, Tel-Aviv University, Tel-Aviv, Israel; John Curtin School of Medical Research, AUSTRALIA

## Abstract

Cell-free protein synthesis (CFPS) systems are important laboratory tools that are used for various synthetic biology applications. Here, we present a simple and inexpensive laboratory-scale method for preparing a CFPS system from *E*. *coli*. The procedure uses basic lab equipment, a minimal set of reagents, and requires less than one hour to process the bacterial cell mass into a functional S30-T7 extract. BL21(DE3) and MRE600 *E*. *coli* strains were used to prepare the S30-T7 extract. The CFPS system was used to produce a set of fluorescent and therapeutic proteins of different molecular weights (up to 66 kDa). This system was able to produce 40–150 μg-protein/ml, with variations depending on the plasmid type, expressed protein and *E*. *coli* strain. Interestingly, the BL21-based CFPS exhibited stability and increased activity at 40 and 45°C. To the best of our knowledge, this is the most rapid and affordable lab-scale protocol for preparing a cell-free protein synthesis system, with high thermal stability and efficacy in producing therapeutic proteins.

## Introduction

Proteins have increasing medical, industrial and research importance, owing to their high versatility, bio-specificity and potency [1]. In accordance, there is a growing need for efficient and economical methods for producing proteins [2–4]. Expressing proteins in yeast, bacteria or mammalian cells is usually the first choice; however, for some proteins this is impossible and other expression systems are needed [5]. Protein synthesis in a cell free system can allow the production of toxic proteins, the incorporation of artificial amino acids, and high-throughput screening of proteins [6].

**Fig 1:**
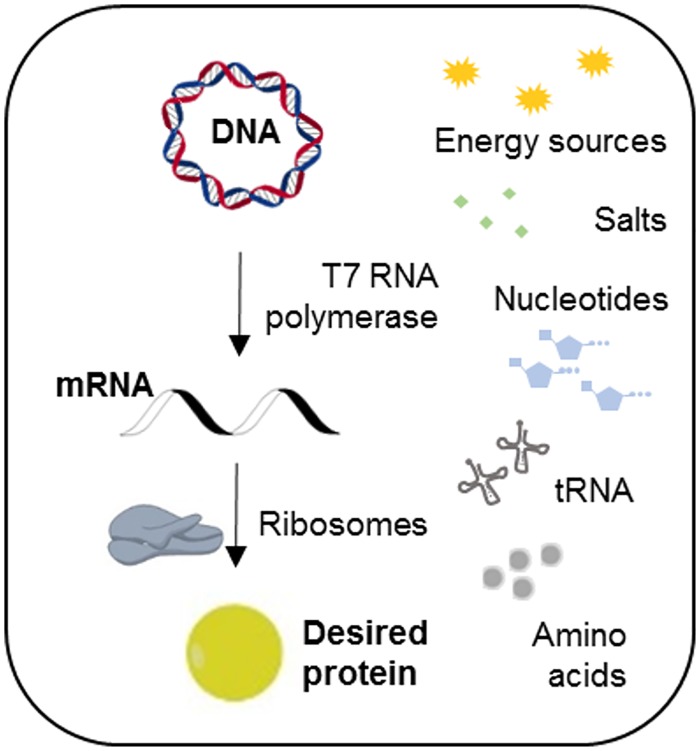
A schematic overview of the cell-free protein production process.

CFPS systems can be prepared from either a cell extract, or from a combination of purified recombinant proteins [7–9]. Currently, CFPS systems based on *E*. *coli* extracts, termed ‘S30 extracts’, are most commonly used and studied. The resulting extract contains all the molecular machineries required for coupling transcription-translation processes, while the obtained protein is encoded by the adddition of a DNA template. For protein synthesis to be efficient, energy sources, amino acids, nucleotides and salts are necessary supplements (Fig 1) [10].

Since first introduced by Pratt and cowokers, the ‘S30 extract’ platform has enabled the expression of a variety of proteins, and its composition has been improved over time [7]. Among the proteins produced with CFPS are antibacterial polypeptides and mammalian membrane proteins, which were challenging to produce in whole cell systems [11–13]. In addition, cell-free systems enable to modify the target protein by introducing disulfide bonds, and incorporating non-natural and isotope-labeled amino acids [7, 8, 14–17]. Though *E*. *coli* extracts are commercially available, they are expensive and sensitive to shipping conditions and freeze-thaw cycles, hindering wide use in academic and research labs.

Due to lengthy procedures and expensive reagents, there has been an emphasis on reducing the cost of CFPS system preparation by optimizing high-speed centrifugation, dialysis and pre-incubation steps [2, 4, 18]. Here, we present a simple, yet efficient, method to produce thermally-stable S30-T7 CFPS systems.

## Materials and Methods

### Materials

The DNA template encoding *Renilla* luciferase was obtained from the S30-T7 high yield protein expression system kit, purchased from Promega (Madison, WI, USA). A *Superfolder* GFP (*sf*GFP) template was purchased from Sandia BioTech (Albuquerque, New Mexico, USA). This DNA template was cloned into a pET9a or a pET28a vector using restriction sites *NdeI* and *BamHI*. A pET9d plasmid encoding Tyrosinase from *Bacillus megaterium* was kindly provided by Prof. Ayelet Fishman [19]. Plasmid pVC45 f+t QQΔ vector was used to produce *Pseudomonas* exotoxin A (PE) [20]. These protein nucleotide sequences are detailed in Appendix A in S1 File.

### Preparation of S30-T7 lysate

A detailed protocol for the preparation of the S30-T7 lysate is described in Table 1. Briefly, S30-T7 lysates were prepared from *E*. *coli* BL21(DE3) and MRE600 transformed with TargeTron^®^ vector pAR1219 (Sigma-Aldrich, Rehovot, Israel). Bacteria glycerol stocks were streaked on an agar Luria Bertani (LB) plate. A single colony was used to inoculate fresh LB media, and was grown overnight at 37°C with orbital shaking of 250 rpm. This was used as a starter to inoculate fresh Terrific Broth (TB) the following day at a 1:50 starter:medium ratio. The media were sterilized and supplemented with ampicillin at 50 μg/ml. The culture was grown at 37°C to OD_600_≈1, upon which 0.4 mM 0.2 μm filtered Isopropyl β-D-1-thiogalactopyranoside (IPTG) solution was added. The culture was further grown at 37°C until it reached OD_600_≈4 and was centrifuged at 7,000 x g for 10 min at 4°C. The pellet was re-suspended in the same volume (1:1 v/v) of cold S30 lysate buffer containing: 10mM Tris-acetate at pH = 7.4, 14mM magnesium acetate, 60 mM potassium acetate, 1 mM 0.2 μm filtered dithiothreitol (DTT) and 0.5 ml/L 2-mercaptoethanol. Subsequently, the suspension was centrifuged again and was resuspended in 15 ml of S30 lysate buffer. Then, the cells were broken by one pass through an emulsiFlex-C3 high pressure homogenizer (Avestin, Mannheim, Germany) that was pre-cooled to 4°C and at a working pressure of 15,000 psi, with an air pressure of 4 bar. One-hundred μl of 0.1 M DTT was added to each 10 ml of homogenized suspension. Finally, the suspension was centrifuged at 24,700 x g or 13,000 x g for 30 min at 4°C divided into aliquots of 200 μl, frozen by liquid nitrogen and stored at -80°C for further use.

**Table 1:** Preparation of S30-T7 lysate.

**A. Materials and solutions required:**	**Notes:**
*E*. *coli* BL21(DE3) or MRE600 transformed with pAR1219	
Ampicillin stock at 50 mg/ml	
LB agar (1.5%) plate	Should contain ampicillin at 50 μg/ml.
LB media (20 ml)	Should be prepared and sterilized in advance. Before bacteria inoculation, ampicillin should be added to final concentration of 50 μg/ml.
TB media (1 liter)	Should be prepared and sterilized in advance. Before bacteria inoculation, ampicillin should be added to final concentration of 50 μg/ml.
100 mM Isopropyl β-D-1-thiogalactopyranoside (IPTG) stock solution	Should be filtered using 0.2 μm filter.
0.1M dithiothreitol (DTT) stock solution	Should be filtered using 0.2 μm filter.
S30 lysate buffer (1.5 liters) containing: 10mM Tris-acetate at pH = 7.4 14mM magnesium acetate 60 mM potassium acetate 1 mM DTT 0.5 ml/liter 2-mercaptoethanol	The S30 lysate buffer was prepared in advance without the addition of DTT and 2-mercaptoethanol, sterilized and stored at 4°C. Prior to use, DTT and 2-mercaptoethanol were added.
Liquid nitrogen	
**B. Equipment:**	**Notes:**
Sterilized Erlenmeyer flasks	2 of 100 ml
Sterilized Erlenmeyer flasks with baffles	2 of 2 liters
Floor incubator shaker	
Centrifuge	Should enable at least 13,000 x g
High pressure homogenizer	Should be pre-cooled to 4°C
-80°C freezer	
Sterilized 1.5-ml plastic tubes	
A spectrophotometer	
Sterilized graduated cylinder	
Sterilized centrifuge tubes	
Sterilized pipette tips	
**C. Procedure:**	
1.1 Streak the bacteria (transformed with pAR1219) on an LB-agar plate. 1.2 Use a single colony to inoculate 10 ml LB media in 100 mL flask, and grow it overnight at 37°C with shaking at 250 rpm on a floor incubator shaker. This step will obtain a starter solution (duplicate). 1.3 Inoculate each one of the 500 ml TB media inside a 2 liters flask using the 10 ml starters. 1.4 Grow the culture at 37°C until it reaches OD_600_≈1, by monitoring the culture’s OD at 600 nm using a spectrophotometer. Once the required OD is achieved, add 0.4 mM IPTG. 1.5 Grow the culture at 37°C until it reaches OD_600_≈4. 1.6 Centrifuge at 7,000 x g for 10 min at 4°C. 1.7 Re-suspend each peleet in 500 ml of S30 lysate buffer, and centrifuge at 7,000 x g for 10 min at 4°C. 1.8 Resuspend in 15 ml of S30 lysate buffer. 1.9 Break the cells by one pass through a high pressure homogenizer at a working pressure of 15,000 psi, with an air pressure of 4 bar. 1.10 Add 100 μL of 0.1 M DTT to each 10 ml of homogenized suspension. 1.11 Centrifuged the suspension at 24,700 x g or 13,000 x g for 30 min at 4°C. 1.12 Divide the supernatant into aliquots of 200 μL into 1.5 ml tubes. 1.13 Freeze the tubes immediately by liquid nitrogen and store at -80°C for further use.	

### *In vitro* protein synthesis using cell-free system based on S30-T7 lysate

A detailed protocol for the *in vitro* protein synthesis reaction is described in Tables 2 and 3. Briefly, The CFPS reaction mixtures were composed of 55 mM HEPES-KOH (pH = 8), 14 mM magnesium acetate, 50 mM potassium acetate, 155mM ammonium acetate, 3% (w/v) polyethylene glycol, 40 mM D-(−)-3-Phosphoglyceric acid disodium salt, 2.5 mM of each natural amino acid (alanine, arginine, asparagine, aspartic acid, cysteine, glutamine, glutamic acid, glycine, histidine, isoleucine, leucine, lysine, methionine, phenylalanine, proline, serine, threonine, tryptophan, tyrosine, valine), 1.2 mM ATP, 1 mM GTP, 0.8 mM UTP, and 10 μg/ml DNA template. The DNA template was transformed to *E*. *coli* BL21(DE3), produced and purified using QIAGEN Plasmid mini and maxi kits (Qiagen, Valencia, CA, USA). In addition, the total protein content of the S30-T7 lysates was measured by Quick Start^™^ Bradford Protein Assay (BioRad) and set to 22 mg/ml. 30% (v/v) of the S30-T7 lysates were added to the reaction mixture. The reaction volume was completed with DNase-, RNase-free water and incubated at a constant temperature of 37°C (unless mentioned otherwise) for 2 hours with vigorous shaking, 1200 RPM. Reagent cost calculations were based on reagent prices obtained from the 2016 online catalogues of Sigma-Aldrich. Cost of labor was not included.

**Table 2:** *In vitro* protein synthesis using cell-free system based on S30-T7 lysate.

**A. Materials and solutions required:**	**Notes:**
1M HEPES-KOH (pH = 8)	
1M magnesium acetate	
1M potassium acetate	
5.2 M ammonium acetate	
50% (v/v) Polyethylene glycol 6000 (PEG)	
0.5 M 3-phosphoglycerate (3-PGA)	
50 mM of 17 amino acids	These 17 amino acids are alanine, arginine, asparagine, aspartic acid, cysteine, glutamine, glutamic acid, glycine, histidine, isoleucine, leucine, lysine, methionine, proline, serine, threonine, and valine.
50 mM of 3 amino acids	These 3 amino acids are tryptophan, phenylalanine, and tyrosine.
100 mM Adenine triphosphate (ATP)	
50 mM Guanidine triphosphate (GTP)	
100 mM Uridine triphosphate (UTP)	
S30-T7 lysate	Prepared according to Table 1
DNase, RNase free H_2_O	
**B. Equipment:**	**Notes:**
Floor incubator shaker or a Thermomixer^®^	
**C. Procedure:**	
1.1 Prepare the CFPS reaction mixtures according to Table 3. Make sure to thaw and add the S30-T7 lystae just prior to the incubation and protien production step. 1.2 Incubate the reaction using a floor incubator shaker at 250 rpm, or a Thermomixer^®^ at 1200 rpm at a constant temperature for 2 hours. When this protocol is first used, it is recommened to incubate the reaction at 37°C. In addition, perform a parallel reaction without DNA, to obtain a negative control. 1.3 Evaluate the produced protein amount using a suitable method, according to the target protein properties.	

**Table 3:** The composition of the CFPS reaction mixture.

Cell-free reaction component	Final concentration in solution
HEPES-KOH (pH = 8)	55 mM
Magnesium acetate	14 mM
Potassium acetate	50 mM
Ammonium acetate	155 mM
Polyethylene glycol (PEG)	3% (v/v)
3-phosphoglycerate (3-PGA)	40 mM
20 amino acids	2.5 mM
Adenine triphosphate (ATP)	1.2 mM
Guanidine triphosphate (GTP)	1 mM
Uridine triphosphate (UTP)	0.8 mM
S30-T7 lysate	30% (v/v)
DNA template	10 μg/mL
DNase, RNase free H_2_O	to reaction volume

The CFPS was used to produce *Renilla* luciferase, TyrBm, *sf*GFP and PE. Luciferase activity was evaluated by the Promega’s luciferase assay system (Madison, WI, USA); luminescence was determined. TyrBm activity was evaluated by initially precipitating insoluble components and then adding 1 mM L-Dopa and 1 mM Cu^+2^ to the reaction tubes. After 1 hour of incubation at 37°C, the absorbance was determined at 475 nm using a plate reader. *Superfolder* GFP *in vitro* production was carried out by incubating the reaction mixtures for 2 hours in different temperatures in the range of 25–50°C. The protein production amount was evaluated according to the fluorescence levels at excitation wavelength of 488 nm and emission of 530 nm. Purified protein was produced and used to correlate between fluorescence levels and concentration (further detailed in Appendix B in S1 File.).

### Cell-free protein synthesis of PE

Western blot analysis was used to verify the *in vitro* production of PE. Five μl of each reaction were mixed with a SDS-PAGE sample buffer (concentrated ×4) and boiled for 10 min at 95°C. The samples were loaded onto a 12% SDS-PAGE gel. Following electrophoresis, the gels were blotted onto nitrocellulose membranes, blocked with 5% nonfat milk powder and probed for 1 hour at room temperature with anti-PE polyclonal antibody (Sigma-Aldrich) diluted by 1:7000. After extensive washes, the blots were incubated with horseradish peroxidase-conjugated anti-rabbit, from goat origin, secondary antibody (GenScript, NJ, USA) diluted to 1:10^4^ and developed with Clarity^™^ Western ECL Blotting Substrate (BioRad). The results were visualized using ImageQuant Las4000 (GE, Sweden).

### PE cytotoxicity assay

The cytotoxicity of PE was determined by a 3-(4,5-dimethylthiazol-2-yl)-2,5-diphenyltetrazolinium bromide (MTT) assay as follows: 1×10^4^ cells/well (200 μl) of 4T1 cell-line were seeded in 96-well plates in a RPMI 1640 medium, supplemented with 10% heat inactivated Fetal Bovine Serum (Biological Industries, Beit-Haemek, Israel), 2 mM of L-Glutamine Solution, 10 U/ml of penicillin G sodium salt and 0.01 mg/ml of streptomycin sulfate for 24 hours at 37°C in a 5% CO_2_ humid atmosphere. Then, the media were replaced with cell-free reactions producing PE or with various concentrations of purified PE. The cells were incubated for 12 hours at 37°C. Growth medium was replaced with fresh media for an additional 24 hours. Finally, the growth medium was vacuum drained and 100 μl/well of 1 mg/ml MTT reagent was added. Following a 2 hours incubation at 37°C, 100 μl/well of the MTT extraction buffer (containing: 10% Triton X-100, 0.1N HCl and isopropanol) were added and incubated overnight at 37°C. Cell viability was calculated from the absorbance values read at 570 nm and 690 nm (blank). The percentage of living cells was calculated with respect to the untreated controls that were processed simultaneously.

## Results and Discussion

CFPS systems are used for a variety of synthetic biology applications, including protein evolution and high-throughput screening (Fig 1). To incorporate these systems as routine laboratory techniques, they should be rapid, simple and affordable [4, 21]. Here we present a S30-T7 CFPS system which can be prepared at a laboratory-scale, requiring minimal preparations and materials.

**Fig 2:**
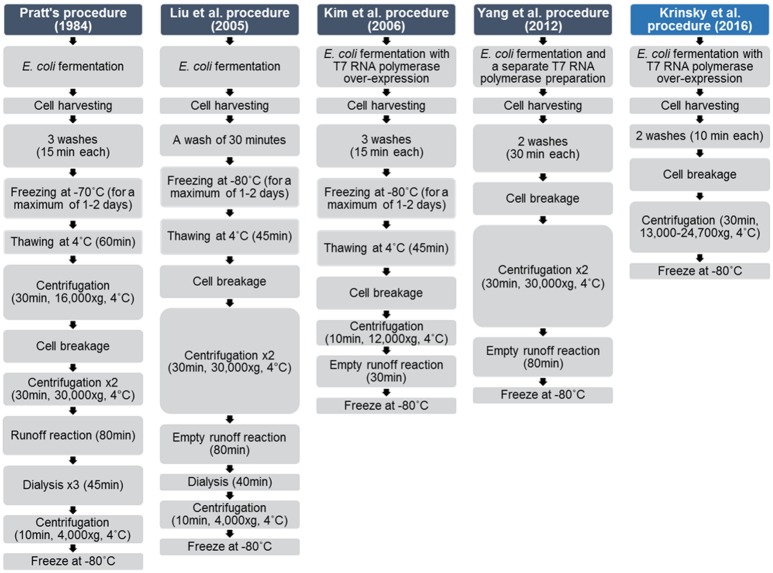
A historical overview of improvements made to CFPS procedures over time.

Since first introduced by Pratt [22], S30 system preparation protocols have undergone several modifications (Fig 2). Previous studies investigated the optimization of the reaction itself; incorporating different energy sources or maintaining magnesium concentration during the reaction [3, 13]. Others have focused on the lysate preparation procedure, which is time consuming with high costs, requiring special laboratory equipment [10, 22]. For example, Liu *et al*. presented a shortened protocol, with reduced cost of the extract preparation by performing an empty runoff procedure (incubation of the lysate in order to degrade endogenous mRNAs, without addition of other components), shortening the dialysis, and cancelling one wash step [2]. Other modifications include the incorporation of endogenous T7 RNA polymerase, or usage of different cell breakage methods including sonication and biochemical disruption [4, 16, 21–24].

### S30-T7 lysate preparation

Bacteria cells were used to over-express T7 RNA polymerase. The cells were washed with a S30 lysate buffer, cracked using a high pressure homogenizer, and were further centrifuged. Then, the suspension was frozen by liquid nitrogen and stored at -80°C for further use. In our new method, only common equipment was used. Specifically, we used a centrifugation force lower then 30,000X g (13,000–24,700Xg), and additional time consuming steps were omitted, such as freezing the cells after harvesting, centrifugation steps, pre-incubation and dialyses. This new protocol requires less than 1 hour of labor from the point the cells have reached the required mass, which is shorter than the procedures mentioned above. For example, Pratt’s system requires more than 7 hours of preparation and Kim *et al*.s' procedure takes 85 minutes [4, 22].

### Reducing reagent cost

Biological processes in cells have been optimized in an evolutionary manner to serve all of the cell's necessities. However, when selecting a single process, such as protein synthesis, it may be further optimized to serve the sole purpose of this specific process, without the limitation and conditions required from other unrelated processes. Accordingly, to further simplify the CFPS system, less ingredients were incorporated into the reaction mixture, as indicated by Ivanir E. [25]. Specifically, 10 instead of 17 ingredient composed the reaction mixture (Table 3): cyclic-AMP, phosphoenol pyruvate, folinic acid and tRNA were omitted [22, 25]. This resulted in a system that costs 80% less compared to Pratt's procedure: 1.1$/50 μl compared to 5$/50 μl at Pratt's protocol [22]. Thus, an affordable system can be easily prepared and applied to produce a variety of proteins.

### Using the system to produce enzymes, fluorescent and luminescent proteins

**Fig 3:**
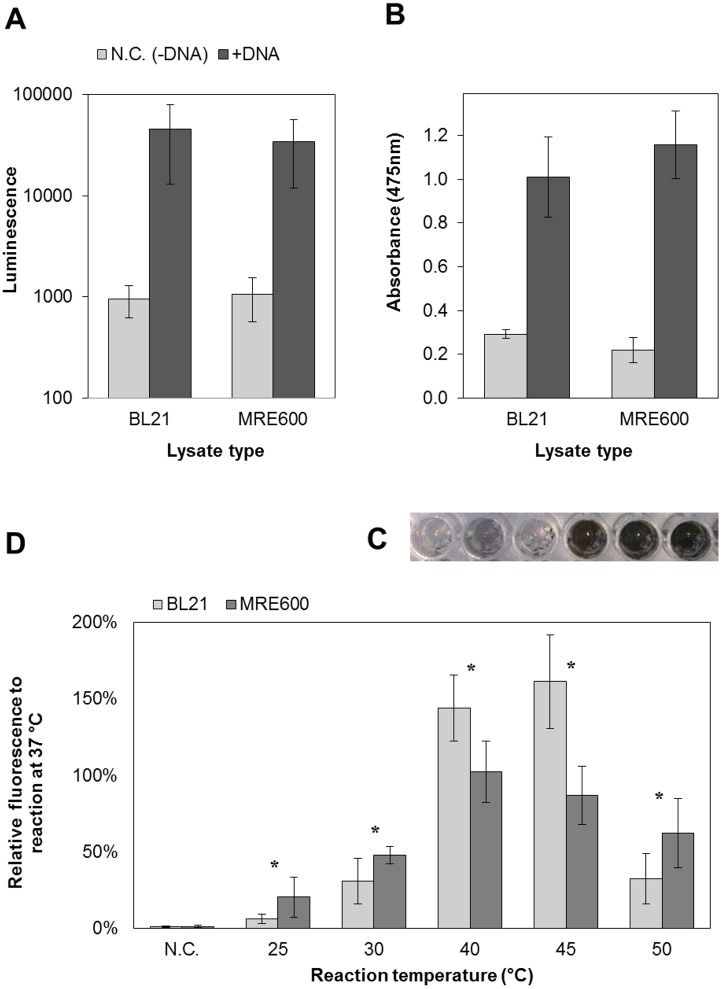
Enzyme productions using S30-T7 CFPS systems sourced from two different *E*. *coli* strains (BL21 and MRE600). (A) The produced *Renilla* luciferase activity was demonstrated by integrating 10 seconds of luminescence measurements (error bars represent standard deviation from at least three independent samples). (B) & (C) TyrBm production was confirmed by monitoring the conversion of 1mM L-Dopa to dopachrome (error bars represent standard deviation from three independent samples). (C) The observed enzymatic activity of TyrBm, produced by the S30-T7 CFPS in a 96-well plate. The three wells to the right present cell-free reaction in the present of DNA template, while in the three wells to the left no DNA template was incorporated into the reaction. The dark color indicates on the conversion of L-Dopa to dopachrome (followed by polymerization and accumulation of melanin), and thus on the production of TyrBm. (D) Temperature effect on cell-free *superfolder* GFP production efficiency of the S30-T7 CFPS (error bars represent standard deviation from at least four independent samples). The protein production amount was evaluated according to the fluorescence levels. The fluorescence values obtained at 37°C were set to 100%, and all the other values were normalized according to them. Negative controls (N.C.) were reactions without DNA templates. * Significant difference between lysates from the two E. coli strains, where α<0.05 according to a Student's t-Test with a two-tailed distribution with equal variance.

Two enzymes were synthesized using the modified S30-T7 system, based on either BL21(DE3) (deficient in the *lon* and *ompT* protease genes) or MRE600 (deficient in ribonuclease I) *E*. *coli* strains. The production of the 36 kDa proteins *Renilla* luciferase (Fig 3A) and TyrBm (Fig 3B) were evaluated according to their enzymatic activity. Both *E*. *coli* strains generated similar protein production. The catalytic reaction of TyrBM was also observed, resulting in a darker solution which indicates on melanin formation (Fig 3C). Different *E*. *coli* strains were previously used to prepare S30 extracts. Among them are BL21(DE3), Rosetta(DE3), BL21-Star(DE3), A19 and C495 [2, 4, 18]. It was found that different preparation conditions are needed to be implemented when preparing extracts from different strains. However, it seems that in the presented S30-T7 CFPS system, when the *in vitro* production was carried out at 37°C, there was no difference between lysates originating from BL21 or MRE600. This system was also used for the production of the 27 kDa fluorescent protein, *sf*GFP (Fig 3D), which can be used as a reporter protein or as a visualization agent [26, 27]. In addition, the reactions were analyzed for the integrity of the produced proteins and for the formation of protein aggregates. It was confirmed that above 95% of the produced protein is the full-length protein however the aggregation propensity is protein dependent (further detailed in Appendixes C, and D and Fig A in S1 File).

### Temperature stability

This system was able to produce up to 150 μg/ml, with variations depended on the plasmid type, produced protein, lysate batch and *E*. *coli* strain. To ensure quantification of a full and active protein, we measured the fluorescent signal from the produced *sf*GFP, and correlated it to the protein concentration. In addition, a fluorescence analysis of *sf*GFP using SDS-PAGE was performed confirming that the fluorescence was obtained from the full length product (further detailed in Appendix E and Fig B in S1 File.). We found that the incubation temperature affects the efficiency of *sf*GFP production (Fig 3D). While previously described CFPS reactions were incubated at 37°C, common for standard protein producing procedures based on *E*. *coli* cells or lysates, we sought to analyze the production of *sf*GFP in a variety of temperatures. BL21-based CFPS system exhibited higher activity in 40 and 45°C, whereas the maximal activity of the MRE600-based system was at 37 and 40°C. Interestingly, the decay in the activity between 45 and 50°C was more drastic for BL21-based system. These findings support former reports that indicate a variability in the nature of CFPS systems, originating from different *E*. *coli* strains due to differences in their genetics [2, 4, 18]. In addition, the high thermo-activity of the presented CFPS system can be implemented in processes which involve high temperatures. For example, if these system are further up-scaled while preserving their properties, they will be less sensitive to transient heat profiles, which may exist in large volume reactors. Another application in the therapeutic field is the production of proteins in an inflammation zone, which is known to involve higher temperature then 37°C [28]. We believe that the increased activity of the bacterial system at temperatures reaching 42°C may be related to the evolutionary survival needs of *E*. *coli* at physiological temperatures of infection and inflammation as well as in conditions of bacterial fermentation [29–33].

### Producing therapeutic proteins with the new S30-T7 system

**Fig 4:**
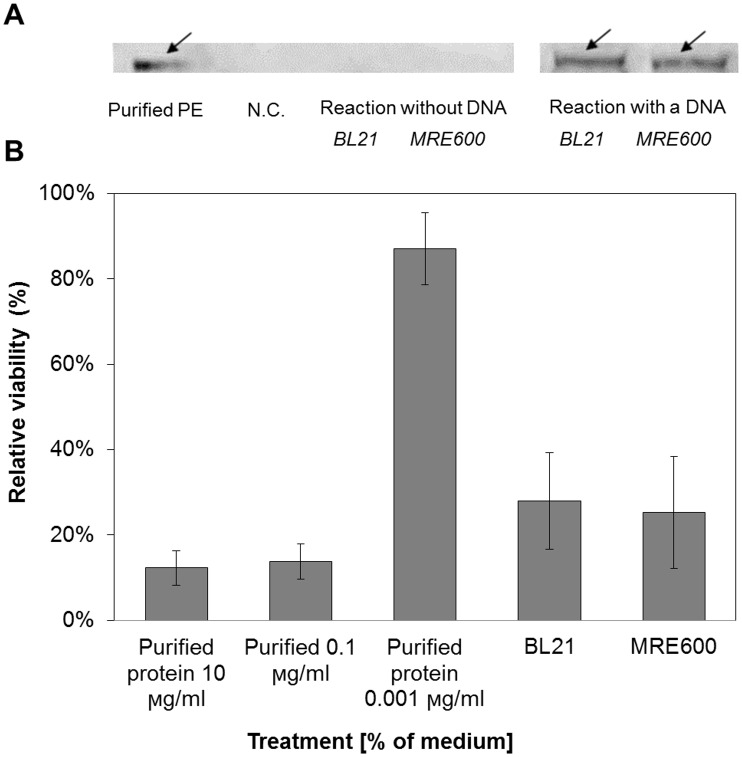
*Pseudomonas* exotoxin (PE) productions using the S30-T7 CFPS system originated from two different *E*. *coli* strains (BL21 and MRE600). Reactions were performed with and without the presence of DNA template. (A) Western blot analysis of cell-free reactions demonstrated the production of PE ~ 66 kDa. Purified PE served as positive control (described in Appendix F in S1 File.). Arrows indicate the position of PE bands. (B) The therapeutic potency of PE was evaluated on 4T1 cell-line. The viability of the cells was determined by MTT assay. Cell viability values obtained without the presence of purified PE or DNA were set as 100%, and the other values were normalized according to them (error bars represent standard deviation from at least three independent samples).

Further demonstration of our CFPS system applicability, was the production of PE, a protein on which many immunotoxins are based [34]. This 66 kDa toxin has been widely investigated for its use in cancer therapy [35]. The *in vitro* production and cytotoxic effect of PE (Fig 4A and 4B) were evaluated. The cell-free reaction resulted in high cytotoxicity on 4T1 cells due to the production of PE. Reactions based on the two lysates did not generate different observations, in compliance with Fig 3A and 3B. The cytotoxicity of a protein produced using a CFPS system against cancer cells has been presented before, however for a lower molecular weight toxic protein (12 kDa) [36]. To our knowledge, this is the first production of a large therapeutic protein using a CFPS system, emphasizing its potential to produce a variety of potent proteins and their incorporation into clinical-related uses.

## Conclusions

We present here a quick, inexpensive and simple procedure for lysate preparation and *in vitro* protein production. This platform was used to synthesize a variety of proteins with molecular weights up to 66 kDa, which possess different functionalities, including cytotoxic activity. The system exhibited improved activity in temperatures higher than 37°C, depending on the bacteria strain, which can be an advantage for different applications.

Development of an affordable, simpler, and more accessible platform can overcome current limitations in protein production and establish a more robust use of CFPS systems, for both research and industry.

## Supporting Information

S1 File Supporting information file contains supplementary figures and appendixes. **Appendix A in S1 File. Protein nucleotide sequences. Appendix B in S1 File. *Superfolder* GFP production and purification. Appendix C in S1 File. Product integrity analysis. Appendix D in S1 File. Analysis of protein aggregation formation. Fig A in S1 File. Protein integrity and aggregation assay.** Cell-free production of *sf*GFP and tyrosinase using the S30-T7 CFPS system (a) & (b) or using a commercial system—the S30 T7 High-Yield Protein Expression System (Promega) (c). The reaction mixtures included biotinylated lysine-tRNA complex, which enables the detection of truncated products. The total and soluble fraction were used to estimate the aggregation formation during the cell-free reactions. **Appendix E in S1 File. Fluorescence analysis of *sf*GFP using SDS-PAGE. Fig B in S1 File. Fluorescence scanning of cell-free reactions.** *Super-folder* GFP was produced by the S30-T7 CFPS system sourced from *E*.*coli* BL21 (a) & (b) or MRE600 (c) & (d). The reaction temperature was 37°C (a) & (c) or 45°C (b) & (d). Each gel was loaded with a protein ladder (lane 1), 3 samples of cell-free reaction containing *sf*GFP encoding plasmid (lanes 2–4), cell-free reaction without a DNA template (lanes 5–7) and purified protein (lanes 8–10 with 3.1 μg, 1.6 μg and 0.8 μg protein, respectively). The primary band indicates that the fluorescence of the functional protein corresponds to the full length product at all reaction conditions. A secondary band is observed due to secondary folding of the protein under mild denaturation conditions. **Appendix F in S1 File. *Pseudomonas* exotoxin A production and purification. Fig C in S1 File. Original Western blot analysis of *Pseudomonas* exotoxin productions.** S30-T7 CFPS system originated from two different *E*. *coli* strains (BL21 and MRE600) and a commercial system (S30 T7 High-Yield Protein Expression System, Promega) were used for the different protein productions. Reactions were performed with and without the presence of DNA template. The yellow frame indicates on the production of *Pseudomonas* exotoxin A. ~ 66 kDa, when a DNA template was incorporated to the reaction. The lower bands are not representing a 66 kDa protein and are related to the S30 extract. They can be contributed to unspecific reactivity of the polyclonal antibodies used in this analysis.
(PDF)
