# Supplementary material for: A Simple and Rapid Method for Preparing a Cell-Free Bacterial Lysate for Protein Synthesis
Source: PLoS One. 2016 Oct 21;11(10):e0165137. doi: 10.1371/journal.pone.0165137 (PMC5074584; doi:10.1371/journal.pone.0165137)
Supplement: S1 File — Appendix A in S1 File. Protein nucleotide sequences. Appendix B in S1 File. Superfolder GFP production and purification. Appendix C in S1 File. Product integrity analysis. Appendix D in S1 File. Analysis of protein aggregation formation. Fig A in S1 File. Protein integrity and aggregation assay. Cell-free production of sfGFP and tyrosinase using the S30-T7 CFPS system (a) & (b) or using a commercial system—the S30 T7 High-Yield Protein Expression System (Promega) (c). The reaction mixtures included biotinylated lysine-tRNA complex, which enables the detection of truncated products. The total and soluble fraction were used to estimate the aggregation formation during the cell-free reactions. Appendix E in S1 File. Fluorescence analysis of sfGFP using SDS-PAGE. Fig B in S1 File. Fluorescence scanning of cell-free reactions. Super-folder GFP was produced by the S30-T7 CFPS system sourced from E.coli BL21 (a) & (b) or MRE600 (c) & (d). The reaction temperature was 37°C (a) & (c) or 45°C (b) & (d). Each gel was loaded with a protein ladder (lane 1), 3 samples of cell-free reaction containing sfGFP encoding plasmid (lanes 2–4), cell-free reaction without a DNA template (lanes 5–7) and purified protein (lanes 8–10 with 3.1 μg, 1.6 μg and 0.8 μg protein, respectively). The primary band indicates that the fluorescence of the functional protein corresponds to the full length product at all reaction conditions. A secondary band is observed due to secondary folding of the protein under mild denaturation conditions. Appendix F in S1 File. Pseudomonas exotoxin A production and purification. Fig C in S1 File. Original Western blot analysis of Pseudomonas exotoxin productions. S30-T7 CFPS system originated from two different E. coli strains (BL21 and MRE600) and a commercial system (S30 T7 High-Yield Protein Expression System, Promega) were used for the different protein productions. Reactions were performed with and without the presence of DNA template. The yellow frame indicates [file pone.0165137.s001.pdf]

SUPPLEMENTARY INFORMATION

A Simple and Rapid Method for Preparing A Cell-Free Bacterial Lysate for Protein Synthesis

Nitzan Krinsky, Maya Kaduri, Janna Shainsky-Roitman, Mor Goldfeder, Eran Ivanir, Itai Benhar, Yuval Shoham and Avi Schroeder*

Outline

Pages 2-4	**Appendix A.** Protein nucleotide sequences.
Page 5	**Appendix B.** *Superfolder* GFP production and purification.
Page 6	**Appendix C.** Product integrity analysis.
Page 6	**Appendix D.** Analysis of protein aggregation formation**.**
Page 7	**Fig A.** Protein integrity and aggregation assay.
Page 8	**Appendix E.** Fluorescence analysis of *sf*GFP using SDS-PAGE.
Page 8	**Fig B.** Fluorescence scanning of cell-free reactions.
Page 9	**Appendix F.** *Pseudomonas* exotoxin A production and purification.
Page 10	**Fig C.** Original Western blot analysis of *Pseudomonas* exotoxin productions.
Page 11	**References**

Appendix A. Protein nucleotide sequences

1. *Tyrosinase* from *Bacillus megaterium* sequence:

ATGGGTAACAAGTATAGAGTTAGAAAAAACGTATTACATCTTACCGACACGGAAAAAAG
AGATTTTGTTCGTACCGTGCTAATACTAAAGGAAAAAGGGATATATGACCGCTATATAGC
CTGGCATGGTGCAGCAGGTAAATTTCATACTCCTCCGGGCAGCGATCGAAATGCAGCA
CATATGAGTTCTGCTTTTTTACCGTGGCATCGTGAATACCTTTTACGATTCGAACGTGAC
CTTCAGTCAATCAATCCAGAAGTAACCCTTCCTTATTGGGAATGGGAAACGGACGCACA
GATGCAGGATCCCTCACAATCACAAATTTGGAGTGCAGATTTTATGGGAGGAAACGGAA
ATCCCATAAAAGATTTTATCGTCGATACCGGGCCATTTGCAGCTGGGCGCTGGACGAC
GATCGATGAACAAGGAAATCCTTCCGGAGGGCTAAAACGTAATTTTGGAGCAACGAAA
GAGGCACCTACACTCCCTACTCGAGATGATGTCCTCAATGCTTTAAAAATAACTCAGTAT
GATACGCCGCCTTGGGATATGACCAGCCAAAACAGCTTTCGTAATCAGCTTGAAGGATT
TATTAACGGGCCACAGCTTCACAATCGCGTACACCGTTGGGTTGGCGGACAGATGGGC
GTTGTGCCTACTGCTCCGAATGATCCTGTCTTCTTTTTACACCACGCAAATGTGGATCG
TATTTGGGCTGTATGGCAAATTATTCATCGTAATCAAAACTATCAGCCGATGAAAAACGG
GCCATTTGGTCAAAACTTTAGAGATCCGATGTACCCTTGGAATACAACCCCTGAAGACG
TTATGAACCATCGAAAGCTTGGGTACGTATACGATATAGAATTAAGAAAATCAAAACGTT
CCTCATAA

A pET9a vector contained this sequence between *Nco* I and *Bgl* II restriction sites [1].

2. *Super folder* GFP sequence:

CATATGAGCAAAGGAGAAGAACTTTTCACTGGAGTTGTCCCAATTCTTGTTGAATTAGAT
GGTGATGTTAATGGGCACAAATTTTCTGTCCGTGGAGAGGGTGAAGGTGATGCTACAA
ACGGAAAACTCACCCTTAAATTTATTTGCACTACTGGAAAACTACCTGTTCCATGGCCAA
CACTTGTCACTACTCTGACCTATGGTGTTCAATGCTTTTCCCGTTATCCGGATCACATGA
AACGGCATGACTTTTTCAAGAGTGCCATGCCCGAAGGTTATGTACAGGAACGCACTATA
TCTTTCAAAGATGACGGGACCTACAAGACGCGTGCTGAAGTCAAGTTTGAAGGTGATAC
CCTTGTTAATCGTATCGAGTTAAAAGGTATTGATTTTAAAGAAGATGGAAACATTCTCGG
ACACAAACTCGAGTACAACTTTAACTCACACAATGTATACATCACGGCAGACAAACAAAA
GAATGGAATCAAAGCTAACTTCAAAATTCGCCACAACGTTGAAGATGGTTCCGTTCAAC
TAGCAGACCATTATCAACAAAATACTCCAATTGGCGATGGCCCTGTCCTTTTACCAGAC
AACCATTACCTGTCGACACAATCTGTCCTTTCGAAAGATCCCAACGAAAAGCGTGACCA
CATGGTCCTTCTTGAGTTTGTAACTGCTGCTGGGATTACACATGGCATGGATGAGCTCT
ACAAAGGATCC

The sequence was cloned into a pET9a or a pET28a vector using restriction sites *NdeI* and *BamHI*.

3. *Super folder* GFP with 6 histidine residues (*sf*GFP-HIS$_6$) sequence:

CATATGAGCAAAGGAGAAGAACTTTTCACTGGAGTTGTCCCAATTCTTGTTGAATTAGAT
GGTGATGTTAATGGGCACAAATTTTCTGTCCGTGGAGAGGGTGAAGGTGATGCTACAA
ACGGAAAACTCACCCTTAAATTTATTTGCACTACTGGAAAACTACCTGTTCCATGGCCAA
CACTTGTCACTACTCTGACCTATGGTGTTCAATGCTTTTCCCGTTATCCGGATCACATGA
AACGGCATGACTTTTTCAAGAGTGCCATGCCCGAAGGTTATGTACAGGAACGCACTATA
TCTTTCAAAGATGACGGGACCTACAAGACGCGTGCTGAAGTCAAGTTTGAAGGTGATAC
CCTTGTTAATCGTATCGAGTTAAAAGGTATTGATTTTAAAGAAGATGGAAACATTCTCGG
ACACAAACTCGAGTACAACTTTAACTCACACAATGTATACATCACGGCAGACAAACAAAA

GAATGGAATCAAAGCTAACTTCAAAATTCGCCACAACGTTGAAGATGGTTCCGTTCAAC
TAGCAGACCATTATCAACAAAATACTCCAATTGGCGATGGCCCTGTCCTTTTACCAGAC
AACCATTACCTGTCGACACAATCTGTCCTTTCGAAAGATCCCAACGAAAAGCGTGACCA
CATGGTCCTTCTTGAGTTTGTAACTGCTGCTGGGATTACACATGGCATGGATGAGCTCT
ACAAAGGAGGGTCCCATCACCATCACCATCACTAAGGATCC

The sequence was cloned into a pET9a vector using restriction sites *NdeI* and *BamHI*.

4. *Pseudomonas* exotoxin A sequence used for cell-free production:

CTAGAAATAAAGAAGGAGATATACCATGGGCAGCAGCCATCATCATCATCATCACAGCA
GCGCCGAAGAAGCTTTCGACCTCTGGAACGAATGCGCCAAAGCCTGCGTGCTCGACCT
CAAGGACGGCGTGCGTTCCAGCCGCATGAGCGTCGACCCGGCCATCGCCGACACCAA
CGGCCAGGGCGTGCTGCACTACTCCATGGTCCTGGAGGGCGGCAACGACGCGCTCAA
GCTGGCCATCGACAACGCCCTCAGCATCACCAGCGACGGCCTGACCATCCGCCTCGA
AGGCGGCGTCGAGCCGAACAAGCCGGTGCGCTACAGCTACACGCGCCAGGCGCGCG
GCAGTTGGTCGCTGAACTGGCTGGTACCGATCGGCCACGAGAAGCCCTCGAACATCAA
GGTGTTCATCCACGAACTGAACGCCGGCAACCAGCTCAGCCACATGTCGCCGATCTAC
ACCATCGAGATGGGCGACGAGTTGCTGGCGAAGCTGGCGCGCGATGCCACCTTCTTC
GTCAGGGCGCACGAGAGCAACGAGATGCAGCCGACGCTCGCCATCAGCCATGCCGGG
GTCAGCGTGGTCATGGCCCAGACCCAGCCGCGCCGGGAAAAGCGCTGGAGCGAATG
GGCCAGCGGCAAGGTGTTGTGCCTGCTCGACCCGCTGGACGGGGTCTACAACTACCT
CGCCCAGCAACGCTGCAACCTCGACGATACCTGGGAAGGCAAGATCTACCGGGTGCT
CGCCGGCAACCCGGCGAAGCATGACCTGGACATCAAACCCACGGTCATCAGTCATCG
CCTGCACTTTCCCGAGGGCGGCAGCCTGGCCGCGCTGACCGCGCACCAGGCTTGCCA
CCTGCCGCTGGAGACTTTCACCCGTCATCGCCAGCCGCGCGGCTGGGAACAACTGGA
GCAGTGCGGCTATCCGGTGCAGCGGCTGGTCGCCCTCTACCTGGCGGCGCGGCTGTC
GTGGAACCAGGTCGACCAGGTGATCCGCAACGCCCTGGCCAGCCCCGGCAGCGGCG
GCGACCTGGGCGAAGCGATCCGCGAGCAGCCGGAGCAGGCCCGTCTGGCCCTGACC
CTGGCCGCCGCCGAGAGCGAGCGCTTCGTCCGGCAGGGCACCGGCAACGACGAGGC
CGGCGCGGCCAACGCCGACGTGGTGAGCCTGACCTGCCCGGTCGCCGCCGGTGAAT
GCGCGGGCCCGGCGGACAGCGGCGACGCCCTGCTGGAGCGCAACTATCCCACTGGC
GCGGAGTTCCTCGGCGACGGCGGCGACGTCAGCTTCAGCACCCGCGGCACGCAGAA
CTGGACGGTGGAGCGGCTGCTCCAGGCGCACCGCCAACTGGAGGAGCGCGGCTATG
TGTTCGTCGGCTACCACGGCACCTTCCTCGAAGCGGCGCAAAGCATCGTCTTCGGCGG
GGTGCGCGCGCGCAGCCAGGACCTCGACGCGATCTGGCGCGGTTTCTATATCGCCGG
CGATCCGGCGCTGGCCTACGGCTACGCCCAGGACCAGGAACCCGACGCACGCGGCC
GGATCCGCAACGGTGCCCTGCTGCGGGTCTATGTGCCGCGCTCGAGCCTGCCGGGCT
TCTACCGCACCAGCCTGACCCTGGCCGCGCCGGAGGCGGCGGGCGAGGTCGAACGG
CTGATCGGCCATCCGCTGCCGCTGCGCCTGGACGCCATCACCGGCCCCGAGGAGGAA
GGCGGGCGCCTGGAGACCATTCTCGGCTGGCCGCTGGCCGAGCGCACCGTGGTGATT
CCCTCGGCGATCCCCACCGACCCGCGCAACGTCGGCGGCGACCTCGACCCGTCCAGC
ATCCCGGACCAAGAACAGGCGATCAGCGCCCTGCCGGACTACGCCAGCCAGCCCGGG
CAACCGCCGCGCGAGGACCTGTAG

A pVC45 f+t QQΔ vector contained this sequence.

5. *Pseudomonas* exotoxin A sequence used for producing the purified protein:

ATGAAAAAGACAGCTATCGCGATTGCAGTGGCACTGGCTGGTTTCGCTACCGTAGCGC
AGGCCGCGAATTTGGCCGAAGAAGCTTTCGACCTCTGGAACGAATGCGCCAAAGCCTG
CGTGCTCGACCTCAAGGACGGCGTGCGTTCCAGCCGCATGAGCGTCGACCCGGCCAT
CGCCGACACCAACGGCCAGGGCGTGCTGCACTACTCCATGGTCCTGGAGGGCGGCAA
CGACGCGCTCAAGCTGGCCATCGACAACGCCCTCAGCATCACCAGCGACGGCCTGAC
CATCCGCCTCGAAGGCGGCGTCGAGCCGAACAAGCCGGTGCGCTACAGCTACACGCG
CCAGGCGCGCGGCAGTTGGTCGCTGAACTGGCTGGTACCGATCGGCCACGAGAAGCC
CTCGAACATCAAGGTGTTCATCCACGAACTGAACGCCGGCAACCAGCTCAGCCACATG
TCGCCGATCTACACCATCGAGATGGGCGACGAGTTGCTGGCGAAGCTGGCGCGCGAT
GCCACCTTCTTCGTCAGGGCGCACGAGAGCAACGAGATGCAGCCGACGCTCGCCATC
AGCCATGCCGGGGTCAGCGTGGTCATGGCCCAGACCCAGCCGCGCCGGGAAAAGCG
CTGGAGCGAATGGGCCAGCGGCAAGGTGTTGTGCCTGCTCGACCCGCTGGACGGGGT
CTACAACTACCTCGCCCAGCAACGCTGCAACCTCGACGATACCTGGGAAGGCAAGATC
TACCGGGTGCTCGCCGGCAACCCGGCGAAGCATGACCTGGACATCAAACCCACGGTC
ATCAGTCATCGCCTGCACTTTCCCGAGGGCGGCAGCCTGGCCGCGCTGACCGCGCAC
CAGGCTTGCCACCTGCCGCTGGAGACTTTCACCCGTCATCGCCAGCCGCGCGGCTGG
GAACAACTGGAGCAGTGCGGCTATCCGGTGCAGCGGCTGGTCGCCCTCTACCTGGCG
GCGCGGCTGTCGTGGAACCAGGTCGACCAGGTGATCCGCAACGCCCTGGCCAGCCCC
GGCAGCGGCGGCGACCTGGGCGAAGCGATCCGCGAGCAGCCGGAGCAGGCCCGTCT
GGCCCTGACCCTGGCCGCCGCCGAGAGCGAGCGCTTCGTCCGGCAGGGCACCGGCA
ACGACGAGGCCGGCGCGGCCAACGCCGACGTGGTGAGCCTGACCTGCCCGGTCGCC
GCCGGTGAATGCGCGGGCCCGGCGGACAGCGGCGACGCCCTGCTGGAGCGCAACTA
TCCCACTGGCGCGGAGTTCCTCGGCGACGGCGGCGACGTCAGCTTCAGCACCCGCGG
CACGCAGAACTGGACGGTGGAGCGGCTGCTCCAGGCGCACCGCCAACTGGAGGAGC
GCGGCTATGTGTTCGTCGGCTACCACGGCACCTTCCTCGAAGCGGCGCAAAGCATCGT
CTTCGGCGGGGTGCGCGCGCGCAGCCAGGACCTCGACGCGATCTGGCGCGGTTTCTA
TATCGCCGGCGATCCGGCGCTGGCCTACGGCTACGCCCAGGACCAGGAACCCGACGC
ACGCGGCCGGATCCGCAACGGTGCCCTGCTGCGGGTCTATGTGCCGCGCTCGAGCCT
GCCGGGCTTCTACCGCACCAGCCTGACCCTGGCCGCGCCGGAGGCGGCGGGCGAGG
TCGAACGGCTGATCGGCCATCCGCTGCCGCTGCGCCTGGACGCCATCACCGGCCCCG
AGGAGGAAGGCGGGCGCCTGGAGACCATTCTCGGCTGGCCGCTGGCCGAGCGCACC
GTGGTGATTCCCTCGGCGATCCCCACCGACCCGCGCAACGTCGGCGGCGACCTCGAC
CCGTCCAGCATCCCGGACCAAGAACAGGCGATCAGCGCCCTGCCGGACTACGCCAGC
CAGCCCGGGCAACCGCCGCGCGAGGACCTGTAGTAA

A pVC45 f+t QQ∆ vector contained this sequence.

Appendix B. *Superfolder* GFP production and purification:

E. coli BL21 transformed with *sf*GFP-HIS_6-pET9a (nucleotide sequence in Appendix A.3) glycerol stock (-80 °C) was streaked on Luria Bertani (LB) plate solidified with 1.5 % Bacto agar, (Acumedia, Neogen Corporation, MI, USA) supplemented with kanamycin at 25 µg ml^{-1} (LB-kan25) to maintain the plasmids. A single colony was used to inoculate fresh LB- kan25 media. The culture was grown overnight at 37 °C while shaking at 250 rpm on a TU-400 incubator shaker (Orbital Shaker Incubator, MRC, Holon, Israel), and the following day it was used as a starter to inoculate fresh Terrific Broth media (supplemented with 25 µg ml^{-1} kanamycin) at 1:50 starter:medium ratio. The culture was grown at 37 °C to OD_{600}≈ 0.8, upon which 0.4 mM of Isopropyl β-D-1-thiogalactopyranoside (IPTG) was added. The culture was further grown overnight at 37 °C and then centrifuged at 8,000 x g for 10 min at 20 °C using MegaFuge centrifuge (Thermo Scientific). The pellet was resuspended in Binding buffer containing 20 mM Tris-HCl (pH 7.4), 500 mM NaCl and 20 mM Imidazole (according to the ratio of pellet originated from 1 L culture at OD_{600}=5 should be resuspended with 15 ml S30 lysate buffer). Then, the cells were broken by ten passes through an emulsiFlex-C3 high pressure homogenizer (Avestin, Mannheim, Germany) that was pre-cooled to 4 °C and at working pressure of 15,000 psi, with an air pressure of 4 bar. Finally, the suspension was centrifuged at 16,000 x g for 20 min at 15 °C. Then the sample was applied to a Ni-sepharose column (HisTrap HP 5ml, GE Healthcare) and purified using fast protein liquid chromatography (FPLC, AKTA, GE). The buffers used for purification were binding buffer, used for equilibration and washing, and elution buffer containing 20 mM of Tris-HCl (pH 7.4), 500 mM of NaCl and 500 mM of imidazole. In order to purify the *sf*GFP, a gradient of the elution buffer from 0-250 mM imidazole over 25 min was used. Eluted protein was collected and dialyzed against phosphate-buffered saline (PBS). All the elution fractions were analyzed using an SDS-PAGE 12% gel and Coomassie blue staining and their protein concentration was measured using Quick Start™ Bradford Protein Assay (BioRad). The fluorescence signals of different protein dilutions were evaluated using a plate reader at excitation wavelength of 488 nm and emission of 530 nm. A correlation between protein concentration and fluorescence was obtained.

Appendix C. Product integrity analysis:

The cell-free reaction products were analyzed for their integrity and the formation of truncated products. Transcend™ tRNA (Promega, Madison, WI, USA), a biotinylated lysine-tRNA complex, was added to the S30-T7 CFPS system sourced from *E. coli* BL21. DNA templates encoding *sf*GFP and tyrosinase were added to the reaction mixtures. After incubation, SDS-PAGE analyses and blotting onto nitrocellulose membranes were performed. Lanes loaded with lysate were used as negative controls. The membranes were blocked by Tris-buffered saline containing 0.5% Tween® 20 and incubated with streptavidin conjugated to horseradish peroxidase (Jackson immunoresearch, PA, USA). The membranes were developed with Clarity™ Western ECL Blotting Substrate (BioRad) and visualized using ImageQuant Las4000 (GE, Sweden). As a positive control, we used a commercial system - the S30 T7 High-Yield Protein Expression System (Promega). Lanes loaded with reaction without DNA were used as negative controls. Band analysis was performed using the CLIQS software (TotalLab Ltd, UK).

Figs A(a), (b) presented single bands, indicating that truncated proteins were not detected. Quantification of the resulting bands confirms that above 95% of the produced protein is the desired protein - *sf*GFP (27 kDa) or Tyrosinase (36 kDa). Fig A(c) presents similar trend for the control commercial CFPS system.

Appendix D. Analysis of protein aggregation formation:

The cell-free reaction products that were used for the protein integrity evaluation (Appendix C), were further analyzed for their soluble fractions using a centrifugation-based protocol [2, 3]. Each sample was centrifuged at 20,000 x g for 30 min at 4 °C using MegaFuge centrifuge (Thermo Scientific). The supernatant was collected and labeled as the soluble fraction. The samples were analyzed as described previously in Appendix C.

By comparing the total to the soluble fraction, Fig A demonstrates that the aggregation propensity is protein dependent. For example, *sf*GFP is produced in a highly soluble form (about 95% is soluble), while tyrosinase has a large insoluble fraction (47%).

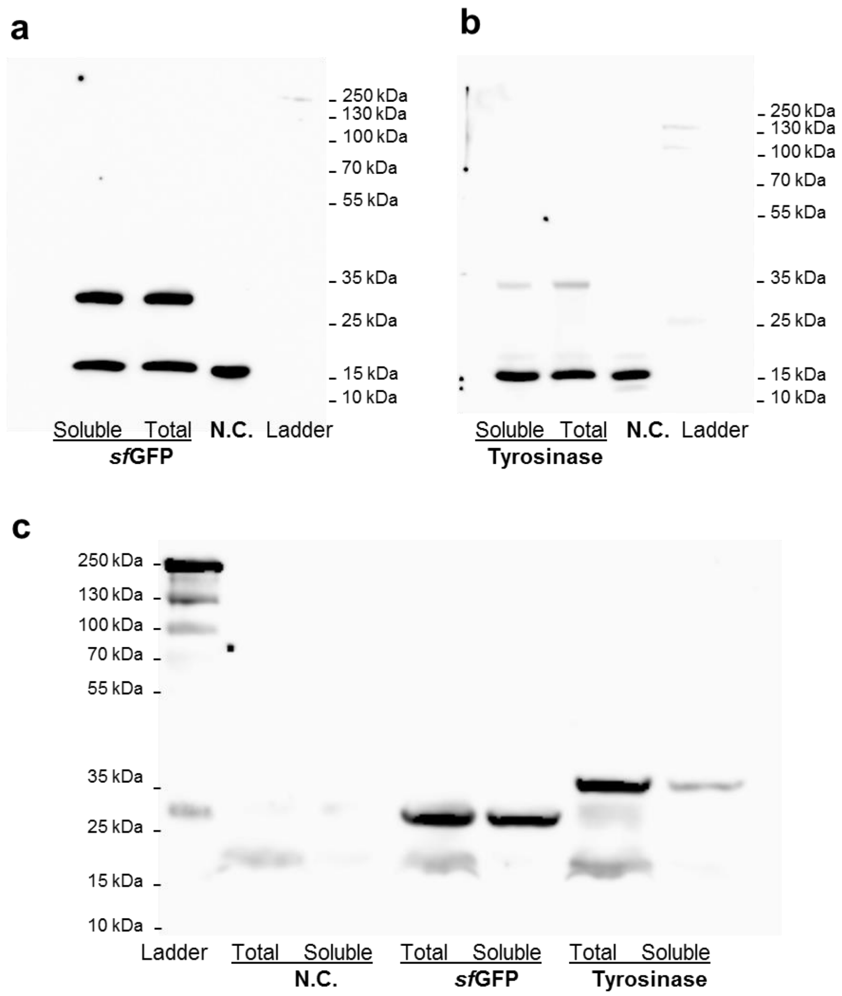

Fig A. Protein integrity and aggregation assay. Cell-free production of *sf*GFP and tyrosinase using the S30-T7 CFPS system **(a) & (b)** or using a commercial system - the S30 T7 High-Yield Protein Expression System (Promega) **(c).** The reaction mixtures included biotinylated lysine-tRNA complex, which enables the detection of truncated products. The total and soluble fraction were used to estimate the aggregation formation during the cell-free reactions.

Appendix E. Fluorescence analysis of *sf*GFP using SDS-PAGE:

In order to demonstrate that the measured fluorescence is obtained by the full protein, SDS-PAGE analysis using fluorescent imaging was used. S30-T7 CFPS systems sourced from two strains of *E. coli* at different reaction temperatures (37 or 45 ˚C) were used to produce *sf*GFP. The produced protein was mixed with SDS-PAGE sample buffer and analyzed using electrophoresis on a 12% SDS-PAGE gel. Each gel was loaded with a protein ladder, reactions with or without *sf*GFP encoding plasmid and purified protein in different concentrations. The gels were visualized using ImageQuant Las4000 (GE, Sweden) according to their fluorescence after excitation with blue laser.

Fig B presents the resulted primary bands, indicating that the fluorescence of the functional protein corresponds to the full length product. These results are in agreement with the Transcend™ tRNA analysis (Fig A(a)), where a single band was also obtained without the detection of truncated products. A secondary band is observed due to secondary folding of the protein under mild denaturation conditions [4].

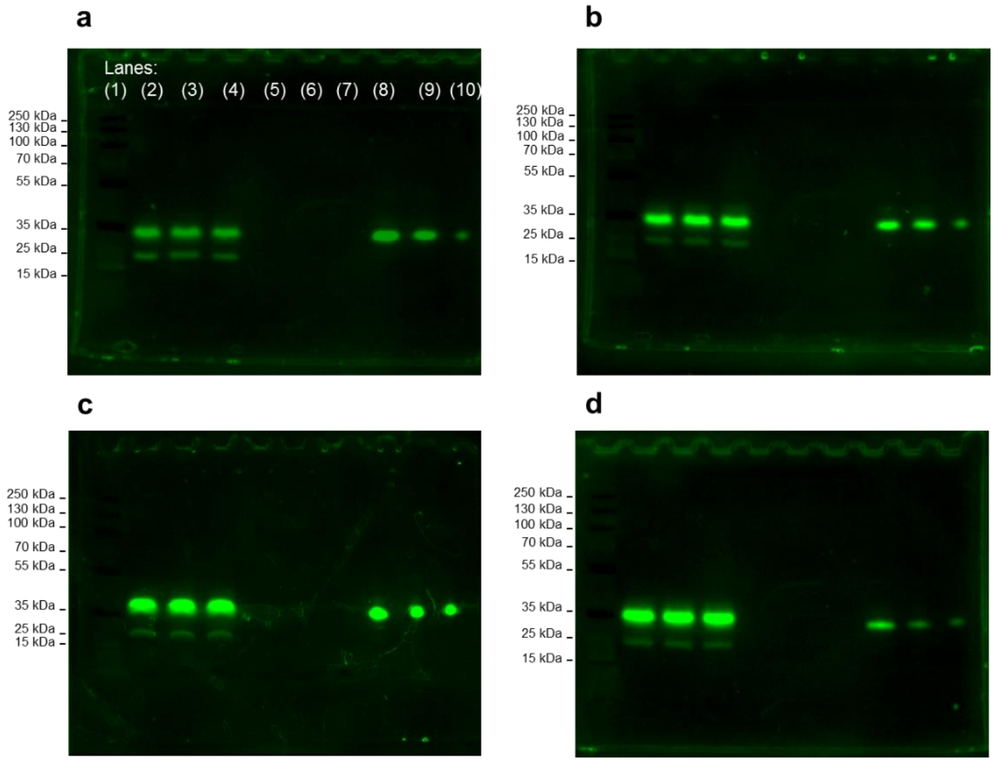

Fig B. Fluorescence scanning of cell-free reactions. *Super-folder* GFP was produced by the S30-T7 CFPS system sourced from *E.coli* BL21 **(a) & (b)** or MRE600 **(c) & (d)**. The reaction temperature was 37 ˚C **(a) & (c)** or 45 ˚C **(b) & (d)**. Each gel was loaded with a protein ladder (lane 1), 3 samples of cell-free reaction containing *sf*GFP encoding plasmid (lanes 2-4), cell-free reaction without a DNA template (lanes 5-7) and purified protein (lanes 8-10 with 3.1 µg, 1.6 µg and 0.8 µg protein, respectively). The primary band indicates that the fluorescence of the functional protein corresponds to the full length product at all reaction conditions. A secondary band is observed due to secondary folding of the protein under mild denaturation conditions [4].

Appendix F. *Pseudomonas* exotoxin A production and purification:

E. coli BL21 transformed with the PE plasmid (nucleotide sequence in Appendix A.5) glycerol stock (-80 °C) was streaked on Luria Bertani (LB) plate solidified with 1.5 % Bacto agar, supplemented with ampicillin at 100 µg ml^{-1} (LB-amp100) to maintain the plasmids. A single colony was used to inoculate fresh LB-amp100 media. The culture was grown overnight at 37 °C while shaking at 250 rpm on a TU-400 incubator shaker (Orbital Shaker Incubator, MRC, Holon, Israel) and used as a starter to inoculate fresh Super Broth media (supplemented with 100 µg ml^{-1} ampicillin) the following day at 1:100 starter:medium ratio. The culture was grown at 37 °C to OD_{600}≈ 2.5, upon which 1 mM IPTG was added. The culture was further grown overnight at 30 °C and then centrifuged at 5,000 x g for 15 min at 4 °C using MegaFuge centrifuge (Thermo Scientific). The pellet was gently resuspended using sterile glass beads in ice cold 20% sucrose, 30 mM Tris-HCl (pH 7.4), 1 mM EDTA (1:5 buffer to original growth media volume), and left on ice for 15 min. Cells were then centrifuged for 15 min at 6000 rpm (FIBRLITE F15-6x100y rotor, Thermo Scientific) at 4 °C. The pellet was gently resuspended in ice cold sterile double-distilled water (1:5 buffer to original growth media volume), and left on ice for 15 min. Following incubation on ice, the periplasmic fraction was collected by centrifuging the cells for 15 min at 7000 rpm and 4 °C (FIBRLITE F15-6x100y rotor, Thermo Scientific). The resulting periplasmic fraction was adjusted to 20 mM Tris-HCl (pH 7.4). The sample was then applied to a Q-sepharose anion exchange column (HiTrap-1ml, GE Healthcare) and purified using fast protein liquid chromatography (FPLC, AKTA, GE). The buffers used for purification were 20 mM Tris HCl pH 7.4 (Buffer A), used for equilibration and washing, and 1 M NaCl in Buffer A (Buffer B), used as the elution buffer. A gradient of Buffer B from 0-100% over 10 min was used in order to purify the PE. Eluted protein was collected and dialyzed against PBS. *E. coli* BL21 that was not transformed with a plasmid was produced and purified as described above and the parallel elution fractions were used as a negative control. All the elution fractions were analyzed using an SDS-PAGE 12% gel and Coomassie blue staining. Proteins eluted after 6 min by the anion exchange column were used as the control for further investigations.

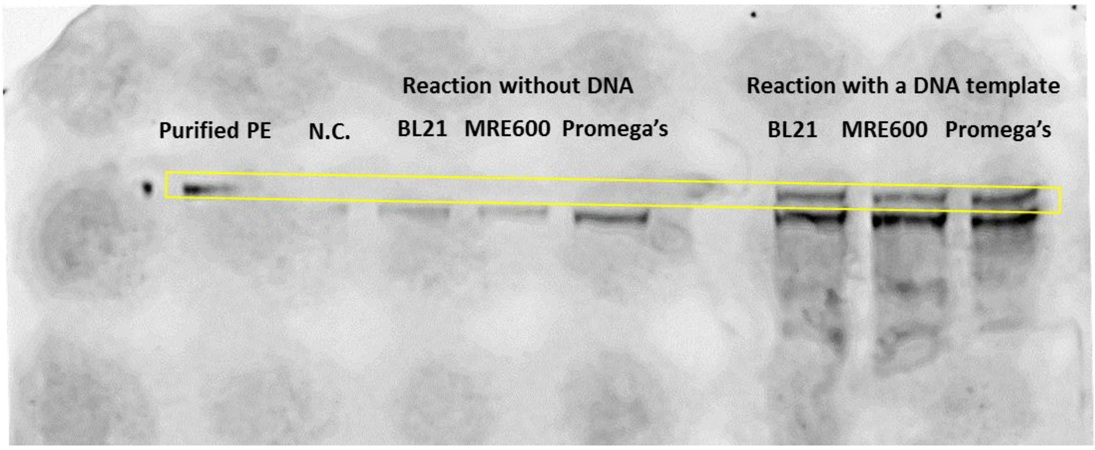

Fig C. Original Western blot analysis of *Pseudomonas* exotoxin productions. S30-T7 CFPS system originated from two different *E. coli* strains (BL21 and MRE600) and a commercial system (S30 T7 High-Yield Protein Expression System, Promega) were used for the different protein productions. Reactions were performed with and without the presence of DNA template. The yellow frame indicates on the production of *Pseudomonas* exotoxin A. ~ 66 kDa, when a DNA template was incorporated to the reaction. The lower bands are not representing a 66 kDa protein and are related to the S30 extract. They can be contributed to unspecific reactivity of the polyclonal antibodies used in this analysis.

References

1. Shuster V, Fishman A. Isolation, cloning and characterization of a tyrosinase with improved activity in organic solvents from Bacillus megaterium. J Mol Microbiol Biotechnol. 2009;17(4):188-200. doi: 10.1159/000233506. PubMed PMID: 19672047.
2. Kang SH, Kim DM, Kim HJ, Jun SY, Lee KY, Kim HJ. Cell-free production of aggregation-prone proteins in soluble and active forms. Biotechnology progress. 2005;21(5):1412-9. doi: 10.1021/bp050087y. PubMed PMID: 16209544.
3. Niwa T, Sasaki Y, Uemura E, Nakamura S, Akiyama M, Ando M, et al. Comprehensive study of liposome-assisted synthesis of membrane proteins using a reconstituted cell-free translation system. Scientific reports. 2015;5:18025. doi: 10.1038/srep18025. PubMed PMID: 26667602; PubMed Central PMCID: PMC4678891.
4. Biyani M, Ichiki T. Solid-Phase Cell-Free Protein Synthesis to Improve Protein Foldability. CELL-FREE PROTEIN SYNTHESIS. 2012:77.
